# Conservative Management of a Massive Retroperitoneal Cyst in Pregnancy: A Case Report

**DOI:** 10.7759/cureus.91108

**Published:** 2025-08-27

**Authors:** Ayano Fujii, Tokumasa Suemitsu, Takahiro Mitani, Mizuho Kadooka, Yoshiaki Furusawa

**Affiliations:** 1 Obstetrics and Gynecology, Kameda Medical Center, Kamogawa, JPN; 2 Obstetrics and Gynecology, The Jikei University School of Medicine, Minato-ku, JPN

**Keywords:** large cyst, mri, parturition disorders, pregnancy, retroperitoneal cyst

## Abstract

Asymptomatic or benign retroperitoneal cysts are typically subjected to observational management. Although rare, cases during pregnancy may present with symptoms such as abdominal pain and distension, or require diagnostic intervention, frequently necessitating surgical procedures. Notably, in pregnant women with cysts exceeding 20 cm, surgical or aspiration procedures are implemented, even if asymptomatic. This report presents a case in which a large retroperitoneal cyst complicating pregnancy was monitored over time using magnetic resonance imaging (MRI), with the patient proceeding to term without symptoms.

A 38-year-old primigravid woman was diagnosed with a uniocular pelvic cyst measuring 20 × 15 × 7 cm by a previous physician, initially suspected to be a para-tubal cyst, which was planned for removal. Intraoperative findings revealed the cyst was of retroperitoneal origin, making it inoperable due to potential intestinal resection. Despite considerations for curative surgery before pregnancy, the patient opted for observational management due to the absence of symptoms and lack of consent. Eight months post-operation, she conceived naturally and consulted our department. Given the cyst's location in the extrapelvic retroperitoneum, it was considered unlikely to obstruct delivery, and conservative management was chosen with surgical intervention as a contingency for symptomatic episodes. Regular MRI evaluations assessed the characteristics and size, which, despite uterine enlargement, were displaced to the right upper abdomen without causing respiratory distress, remained elastic, and showed no signs of rupture or growth. The patient underwent a successful vaginal delivery at 39 weeks of gestation.

In pregnancies complicated by large retroperitoneal cysts, surgical intervention is often considered due to concerns regarding cyst rupture and delivery disorders. However, our case, being benign and asymptomatic, was managed with observation as the definitive surgery would necessitate intestinal resection. This case suggests that retroperitoneal cysts can flexibly adapt to the upper abdomen depending on the site of origin, indicating a low risk of parturition disorders and rupture. This clinical course implies that conservative treatment can be attempted after a thorough assessment of the location and characteristics of the cyst.

## Introduction

Retroperitoneal cysts are located within the retroperitoneal space, without direct connection to other anatomical structures [1]. Diagnosing retroperitoneal cysts is challenging and requires a thorough evaluation of their site of origin. Retroperitoneal cysts originate from various embryological remnants and can be classified into several types, including lymphatic, mesenteric, urogenital, enteric, traumatic, infectious, and neoplastic cysts [2]. This classification is helpful for differential diagnosis and management planning. Additionally, the Losanoff classification, originally proposed for mesenteric cysts, categorizes cysts into four types (I-IV) based on characteristics such as adhesions, malignancy, and complexity. This framework helps guide treatment decisions, especially in distinguishing cases suitable for conservative management from those requiring surgical intervention [3]. While the definitive treatment is surgical resection, conservative management may be an option in cases without malignant features or symptoms [1].

Retroperitoneal cysts in pregnancy are rare, and previous reports indicate that surgical or aspiration procedures have been performed in most pregnancy cases involving cysts larger than 20 cm, even in asymptomatic cases [4,5]. The incidence of retroperitoneal cysts during pregnancy is unknown, as only case reports exist. A 2009 review identified seven cases of cystic lesions in pregnancy (including ovarian and mesenteric cysts), and a 2022 review summarized 17 cases [6,7]. These numbers underscore the exceptional rarity of cystic lesions diagnosed during pregnancy and the limited available data to guide management.

This paper presents a case in which a large retroperitoneal cyst complicating pregnancy was monitored over time via magnetic resonance imaging (MRI), proceeding to term without symptoms. Given the highly invasive nature of the surgical treatment, the patient declined this intervention. By evaluating the cyst’s site of origin, it was determined that the risk of acute abdomen due to torsion or other complications was low, leading to the decision for conservative management.

## Case presentation

A 38-year-old primigravid woman with a BMI of 26 was incidentally diagnosed with a large pelvic cyst at the Department of Reproductive Medicine. Although she was asymptomatic with no abdominal pain, gastrointestinal symptoms, or urinary symptoms, surgery before pregnancy was recommended due to concerns about miscarriage, preterm birth, cyst rupture, and symptom development with uterine enlargement [7]. MRI revealed a cyst measuring 20 × 16 × 7 cm, characterized by hypo-intensity on T1-weighted and marked hyper-intensity on T2-weighted images. Both ovaries were normal, but cyst continuity was unclear. The preoperative diagnosis was a right para-tubal cyst, and a laparoscopic cystectomy was scheduled.

**Figure 1:**
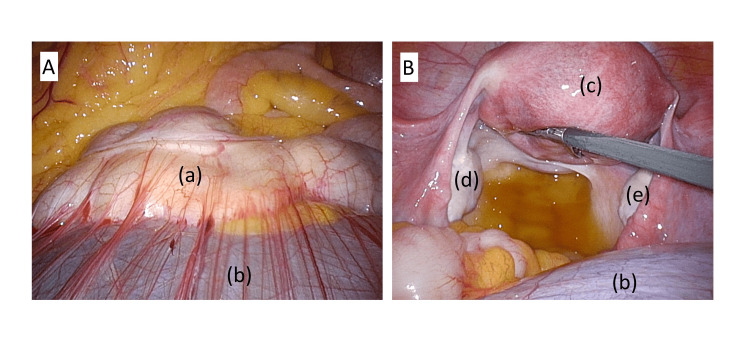
Laparoscopic surgical findings A: The preoperative diagnosis was a para-tubal cyst. However, inoperative findings revealed it to be a retroperitoneal cyst. The cyst is adherent to the transverse colon. B: Both adnexa are normal. (a) Transverse colon (b) Retroperitoneal cyst (c) Uterus (d) Left ovary (e) Right ovary.

Intraoperative findings showed no continuity between the cyst and the adnexa, with adhesions to the transverse colon and pelvic wall, suggesting a retroperitoneal origin (Figure 1). Due to the potential need for intestinal resection, complete cystectomy was deemed challenging. Consequently, an exploratory laparotomy was performed, which revealed no evidence of malignancy in the ascitic fluid. Despite the initial recommendation for definitive surgery pre-conception, the patient opted for conservative management given her asymptomatic status and concerns regarding the invasiveness of intestinal surgery.

**Figure 2:**
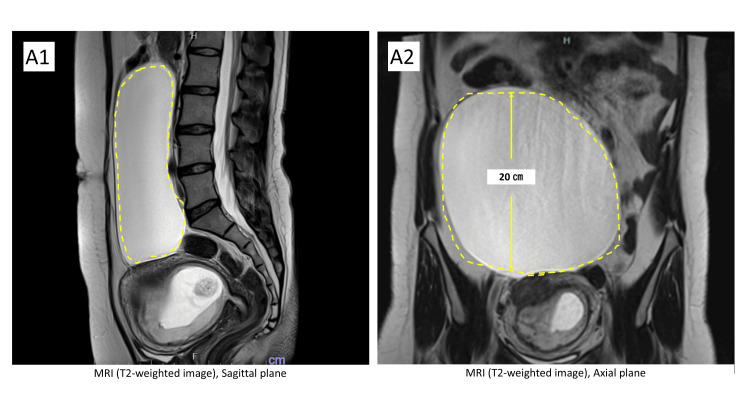
MRI at 11 weeks’ gestation A1.2: The initial MRI image shows a cyst measuring 20 cm in length located above the uterus.

Eight months post-operation, she conceived spontaneously and consulted our department. Large cysts complicating pregnancy are rare and their management and outcomes remain largely unknown. Potential complications include miscarriage, preterm birth, pressure symptoms (such as respiratory distress and abdominal fullness), cyst rupture, associated infections, and acute abdominal pain, which may require emergency surgery or preterm birth [7]. Although puncture aspiration was an option, it could become more difficult as the gestational uterus increased in size. These risks were explained to the patient, and she requested to continue her pregnancy under conservative management. We used MRI to assess the appearance and size of the cyst and to rule out malignancy. The first MRI was performed at 11 weeks of gestation (Figure 2), and the cyst’s location in the extrapelvic retroperitoneum was deemed to pose a low risk of obstructing delivery. 

**Figure 3:**
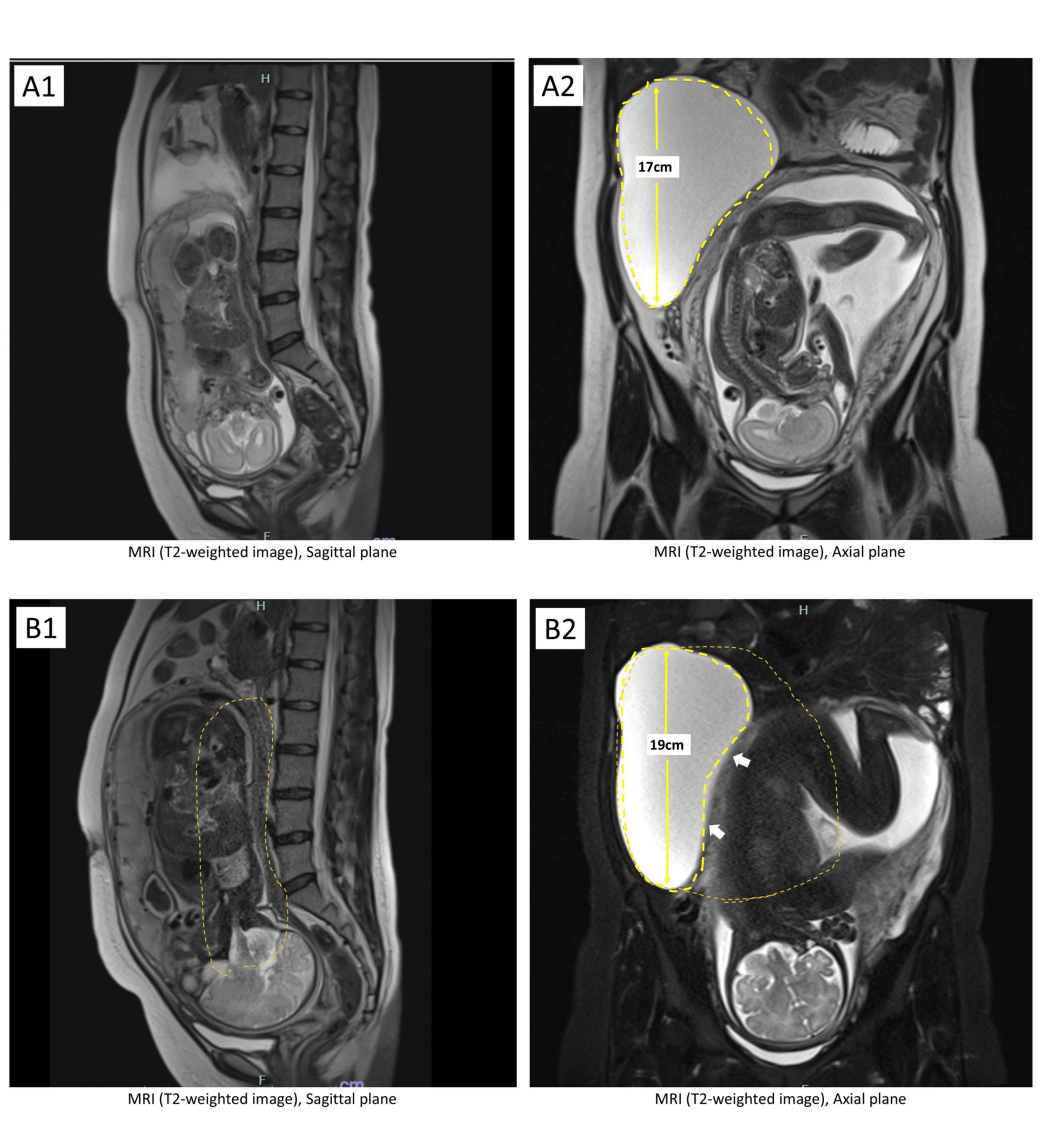
MRI at 25 and 34 weeks’ gestation A1.2:  MRI at 25 weeks’ gestation. The cyst is flexibly displaced to the right upper area of the uterus without significant changes in size. B1.2:  MRI at 34 weeks’ gestation. There is no abdominal distension or respiratory distress, and the cyst size remains unchanged.

**Figure 4:**
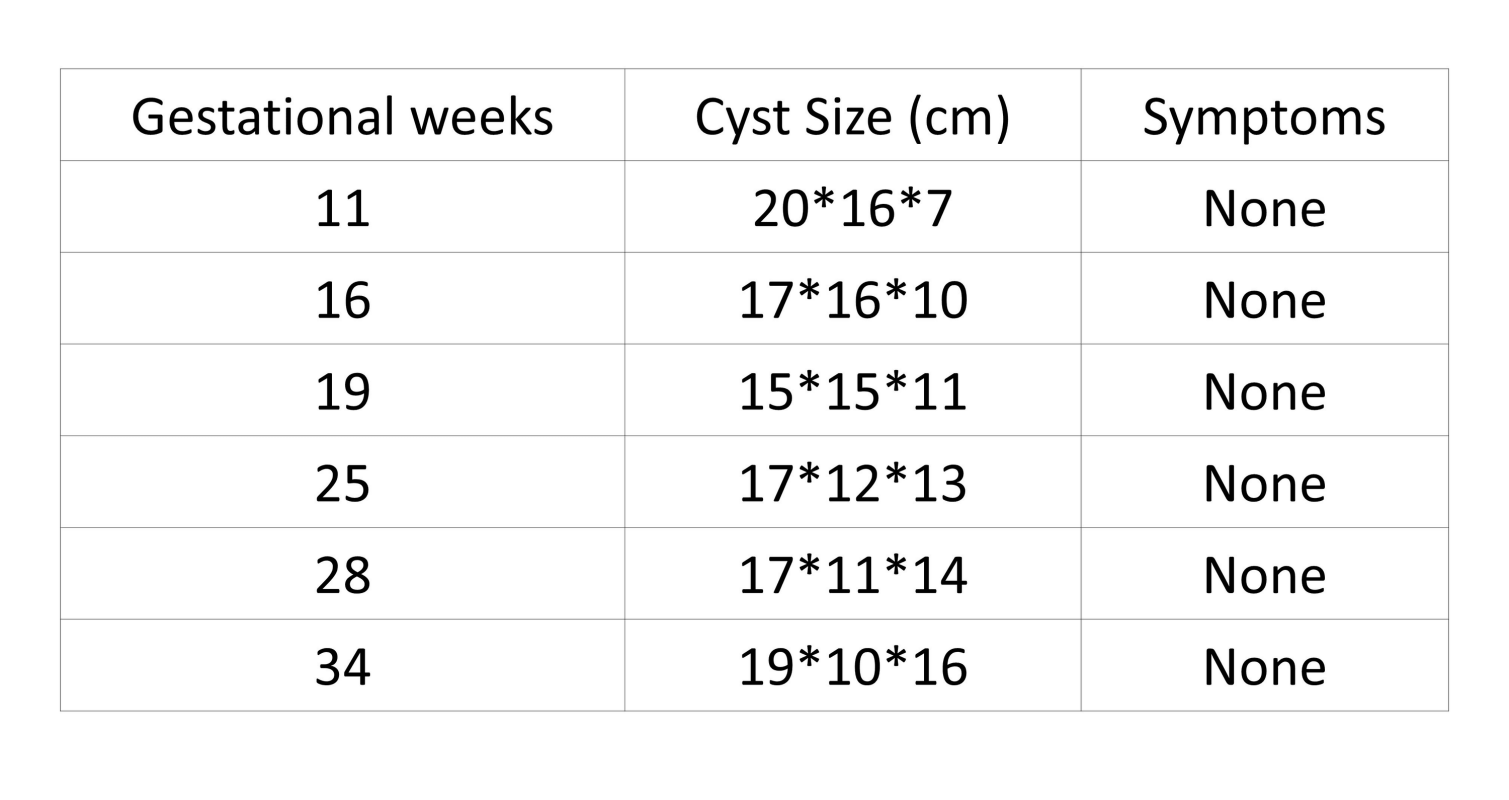
Summary of cyst size and symptoms during pregnancy Follow-up MRI was performed at 11, 16, 19, 25, 28, and 34 weeks of gestation. The cyst showed no significant change in dimensions and remained asymptomatic throughout pregnancy.

With the patient's consent, conservative management was chosen, with surgical intervention as a contingency for symptomatic episodes. Subsequent MRI at 16, 19, 25, 28, and 34 weeks of gestation showed that the cyst deformed flexibly to fit the peritoneal cavity and shifted to the right upper quadrant while remaining stable in size without rupture or significant symptoms (Figures 3, 4).

At 39 weeks 0 days’ gestation, the patient was admitted due to a rupture of the membranes. Epidural anesthesia was administered, but contractions weakened, and oxytocin was initiated. Finally, forceps delivery was performed due to the indication of a prolonged second stage of labor. Uterine fundal pressure was avoided to mitigate the risk of cyst rupture. The neonate, a girl weighing 3,042 g, had Apgar scores of 8 and 9. Arterial blood gas analysis showed a pH of 7.259. The patient recovered well without cyst rupture and was discharged on the fifth day post-delivery.

## Discussion

Information on retroperitoneal cysts during pregnancy is limited. In previous reports, surgical or aspiration procedures were performed in cases with cysts larger than 20 cm, even in asymptomatic cases, and the timing of these interventions varied among cases. This case features a large retroperitoneal cyst identified during pregnancy that was monitored over time via MRI and managed conservatively. Retroperitoneal cysts are located within the retroperitoneal space without a direct connection to other anatomical structures [1]. Differential diagnoses for large cysts include mesenteric, lymphatic, enteric, urogenital, traumatic, infectious, and ovarian cysts [1,8,9]. Where the origin is unclear, some cases have been reported in which the cyst was judged to be of ovarian origin, as in this case, where surgery was performed for a para-tubal cyst at the time of initial diagnosis [4]. Approximately half of the cases present with symptoms, such as abdominal pain, although some instances are incidentally detected [1].

Surgical resection is the definitive treatment for retroperitoneal cysts, and the final diagnosis is based on the site of origin and the pathological results [1]. If imaging shows no malignant features and the patient is asymptomatic, conservative management may be considered depending on the accuracy of the radiological diagnosis. While some cases remain asymptomatic, there are reports of cases requiring emergency surgery due to an acute abdomen. According to a report by Kurtz et al., retroperitoneal cysts, when compared to mesenteric cysts considered in the differential diagnosis, have fewer reports of acute abdomen and related emergency surgeries or intestinal resections [2]. On the other hand, the success rate for complete resection is low, and the recurrence rate is high [2]. This is due to the proximity of the cysts to blood vessels and multiple organs, making surgery difficult. In cases where conservative management is employed, it is imperative to inform the patients that emergency surgical intervention may become necessary in the event of an acute abdomen caused by rupture or torsion. Furthermore, surgical treatment itself carries potential risks, including intestinal resection, which should be explained thoroughly.

A search for pregnancy cases using keywords such as retroperitoneal cyst, mesenteric cyst, and lymphatic cyst revealed only a limited number of reports, and there is no established management protocol for such cases. Most cases involved some form of intervention, even if asymptomatic, due to fetal evaluation challenges [6,7,10,11]. In previous reports, pregnancies complicated by cysts larger than 20 cm were managed with puncture aspiration or surgery [4,5]. However, this case highlights the feasibility of conservative treatment. Emergency surgery during pregnancy carries risks; therefore, many cases involve preventive interventions. In previous reports, most symptomatic patients who underwent emergency surgery had mesenteric cysts originating from the small intestine, and intestinal necrosis was observed [12,13]. These reports suggest that torsion should be considered, depending on the site of origin.

The initial diagnosis in this case was a para-tubal cyst. However, during the surgery, the ovaries and fallopian tubes appeared normal, and the lesion was identified as a retroperitoneal cyst merging with the transverse colon. Torsion is thought to be less likely to occur in cases of retroperitoneal origin, like this one, than in cysts originating from the intestine; therefore, conservative treatment was considered a viable option. Follow-up MRI assessments of the cyst showed that as the pregnant uterus enlarged, the cyst was flexibly displaced upward without causing any bloating or abdominal pain, and progressed to full-term pregnancy without complications. In this case, the cyst was located laterally above the uterus, and its position did not obstruct vaginal delivery, which was another reason for selecting conservative treatment.

Cysts larger than 20 cm are difficult to assess comprehensively by ultrasound. MRI is therefore essential in the early stages of pregnancy to explore the option of conservative management. Furthermore, as confirmed in this case, the cyst was displaced as the pregnant uterus expanded. Therefore, MRI can be valuable in determining the feasibility of vaginal delivery without causing delivery complications. If the cyst is located in a position that may cause delivery complications, puncture aspiration, or other procedures become potential treatment options. Considering the risk of preterm birth associated with these procedures and the potential for cyst recurrence due to fluid re-accumulation, therapeutic intervention after the third trimester is preferable. Consequently, we propose reassessment during late pregnancy. Post-delivery, the patient did not wish to undergo aggressive treatment and remained asymptomatic, so follow-up observation has been continued.

## Conclusions

Surgical treatment for retroperitoneal cysts is challenging due to high invasiveness and potential complications, including intestinal resection. During pregnancy, some patients prefer conservative management to avoid unnecessary surgical risks. While surgical intervention has traditionally been the primary approach, appropriate evaluation of the cyst’s site of origin may allow for conservative management in select cases. In this case, several features supported conservative management, including the large but stable size of the cyst, absence of symptoms, lack of malignant imaging features, and displacement away from the uterus. Given the limited number of reports on pregnancies complicated by cysts, further accumulation of cases is necessary to establish evidence-based management strategies.
